# From Our Correspondents

**Published:** 1983-04

**Authors:** 


### From Our Correspondents

### Return to Traditional 'Final' Examination

In the spring a student's fancy sadly turns to thoughts of toil, specifically the June Finals for the degree of MB ChB (Bristol). Nowadays nearly half our Bachelors of Medicine and Surgery are in fact spinsters, or should I say bachelor girls or even Mss? ("Alas... that Youth's sweet-scented Manuscript should close": Fitzgerald, Omar Khayyám). For 15 years or so the University of Bristol has held an 'integrated' examination for the Final MB ChB. This bold educational experiment ended in December 1982, its passing largely unremarked and for my part unlamented. The return to a traditional type of examination will not jeopardise the arrangement by which written papers are set at the end of the second (clinical) year and a clinical examination at the end of the third year.

The old-style Finals were based on the philosophy that an embryo doctor need merely demonstrate adequate competence at handling patients in any two separate disciplines for it to be assumed that he or she is generally fit to practice; also that in real life patients do not come neatly packaged as 'surgical', 'medical' or whatever. The upshot was that a student might encounter one 'long case' in mental health, for example, plus two or three 'short cases' in perhaps surgery or obstetrics and thereafter be judged proficient to take front-line responsibility for a ward full of medical patients. Clearly any examination is arbitrary, and some would argue that real knowledge can only be acquired after qualification and under proper supervision. Nevertheless, most teachers and many students felt cheated by such a superficial test.

There were certain palpable absurdities under the old system. By dodging the surgical essay question and missing a surgical clinical, a student could acquire a ChB degree on the basis of 40 multiple choice questions (MCQ's) in surgery. Because they were optional, neither the essay nor the clinical could be taken into account in selecting candidates for a distinction viva.

Two 'continuous assessment' gradings were taken into account, but gradings vary from firm to firm and remain widely distrusted by the student body. Six centres in Bristol were needed to run the examination, each requiring a mixture of patients, including children, pregnant women and the mentally ill. Each centre had one external examiner, who remained aloof from the bread-and-butter examining but would be called upon to take any candidate in peril of failure for an extra viva, quite possibly on cases outside his own discipline.

What lies ahead is a resumption of the status quo, with students being required to pass separate exams in each of the major clinical subjects. These marks can now be taken into consideration for a distinction in any one component discipline. There are new logistic problems, but also benefits. In surgery it means a contraction from six centres to two but an expansion from one to two days and from one to four external examiners; each student will encounter one external.

The University's intention is not to deprive the South West of preregistration houseman but to ensure that their clinical assessment is more just and less quixotic. Narrow failure in any one subject will be recoverable by an overall pass-mark in the whole examination. Irredeemable failure will require the candidate to re-sit that subject at least. The revised hurdle should hold no terrors for the vast majority of students, who have survived a rigorous selection procedure to reach this stage of their careers. One consequence is certain: the results of our surgical teaching, previously in the penumbra, will be exposed to the harsh light of mid-summer.

R.C.N.W.

### "Death Watch" Meetings

One of the unique features of the Southmead Hospital Medical scene is the Clinical Staff Meetings. These have taken place regularly during term time on the evening of the fourth Wednesday of each month for the past 32 years and are locally known as the Death Watch Meetings.

The most recent meeting, held on January 25th 1983 was memorable in many ways, chiefly in that Dr Norman Brown was in the Chair. This was quite fortuitous since, as the two clinical chairmen were unable to be present on this evening, he had kindly agreed to "stand in". By tradition the chairmen have always been clinicians. Dr Brown F.R.C.P. could not be excluded on these grounds and the evening was memorable since not only did Norman Brown start these meetings in 1950, act as secretary for 25 years, but sadly he retires at the end of March.

In a brief and timely introduction to the evening's proceedings Dr Brown reminded the large audience of the origins of this peculiarly Southmead meeting. Prior to the first meeting, held on Hallow'en 31.10.1950 under the chairmanship of Professor A. V. Neale, the consultant staff at Southmead had been circulated to 'obtain their consent for patients dying under their care to be discussed at these meetings'. With one exception all consultants had agreed. The format and content of these meetings has changed over the years. During the 1950's up to 12 cases would be discussed in one evening with up to 38 consultants in attendance. Originally confined to consultants, the junior staff were invited after 1953 and the Ham Green staff joined in 1956. By the 1960's the current pattern of discussing four deaths of the previous month was established. Dr Brown also reminded the audience that the present Editor of this Journal, Mr Michael Wilson, on taking the Chair in 1960, introduced the excellent tradition of providing port, which, together with coffee, adds a certain something to these discussions concerning death.

It is also relevant that these meetings are held in a Committee room and not in a lecture theatre. This 'around the table' arrangement in some subtle way seems to eliminate the 'them and us' atmosphere that can develop in similar meetings in other hospitals, both between the pathologists and clinicians and between rival clinical specialities. The projection equipment is rather primitive but an X-ray viewing box is available. The 'denouements' of the radiologist (often seeing the pictures for the first time) have provided frequent memorable moments. Another tradition of 'passing the meat' around on trays might disturb the sqeamish, but this is softened by the port and does indeed reflect the 'Morbid' nature of the evening's proceedings.

Only once has a psychiatrist been directly involved in the meetings concerning a patient with Anorexia nervosa? Thyrotoxicosis; but the philosophical, psychological as well as the histopathological aspects of death are frequently pursued. Choosing the best cases for discussion is still not easy. A patient who had been seen by both physicians and surgeons, with good clinical and investigative data, in whom the necropsy revealed the unexpectd, is a 'sure winner', but it is amazing how often the 'quicky' produces the most stimulating discussion. May this most admirable form of 'Medical Audit', started by NJB so long ago, continue to educate, illuminate and entertain the Southmead medical staff as it has done for so many years without rancour or ill-will.

C.R.T.

**Paying for it All**

Now that the industrial troubles are over in the N.H.S., we can take stock of our present position, and immediate prospectus. Most clinics are running normally and the wards are as full as ever. The major problem is in planning for new developments, or even replacing worn out equipment. Most districts are overspent on medical and surgical equipment (Southmead to the extent of £120,000, Bristol and Weston £160,000). How best can we acquire for example a new ultrasound apparatus, a replacement electrocardiograph or a new dialysis facility?

The public seems increasingly keen to contribute to new developments and research, and large sums have been collected for kidney machines, kidney research and patients' comforts. Considerable sums have been collected for intensive care equipment in all Bristol hospitals, either from grateful patients or established charities such as the British Heart Foundation, local Lions, Round Table, etc. We are most grateful to all these contributors, who enable developments to occur and release exchequer funds for the less glamorous building, plumbing and decorating.

Medical staff could, however, make a greater contribution to the financial problems of the districts. If we really want to see better facilities for cardiac surgery and hip replacements, to take two examples, we must economise elsewhere. Do we really need to spend 10% more on pathology tests each year? Instead of ordering all the tests that might be useful, should we not concentrate on those few which have a specific or discriminating value? Cannot more of our surgical procedures be done as out-patients or in a five day ward, thus achieving great economies in nursing costs and at the same time improving patients contentment? Can we not reduce out-patient follow-up in all disciplines thus freeing medical and nursing staff for other duties?

It is my belief that we cannot expect any significant increase in public money during the present recession, and that the profession must show leadership and a sense of responsibility by economising whenever possible in order that developments can occur.

H.G.M.

**Tulip Trees from Pennsylvania**

We were fortunate recently at Frenchay Hospital to have had the opportunity to be involved in the continued historic link between the American State of Pennsylvania and Frenchay Village.

It might be helpful to recall that in 1681, William, the son of one of Charles II's great Admirals, Sir William Penn, whose arms and armour hang high on the wall of St. Mary Redcliffe Church, was granted a Lease of land beyond the Delaware in payment of Royal debts. The land was named appropriately as Pennsylvania, and William was nominated as first Governor. Whilst at Oxford as a young undergraduate he had become a Quaker, and at that time Frenchay Village and its environment had become home to several famous Quaker families, and the Quaker Meeting House (1673, rebuilt 1809) used then, still stands now and remains in active use. In 1696 William Penn married Hannah Callowhill who lived at Frenchay, at the other Friends Meeting House, now part of Quakers' Friars.

To commemorate these close associations two tulip trees were sent from Pennsylvania at that time and planted in the grounds of two of Frenchay's houses. They both thrived and became very large, lovely trees, but unfortunately age and disease recently overtook them, particularly one of them in the garden of Mr. Robert Woodward.

As a result of the contact he made with the American Embassy in London, the State of Pennsylvania very generously sent another six young Tulip trees (Liriodendron tulipifera), and Turner L. Oyloe, Counsellor for Agricultural Affairs at the American Embassy came down to Bristol on January 27th 1983 to perform the planting ceremony. Two of these trees were planted in the grounds of Frenchay Hospital, at a splendid ceremony presided over by the Chairman of the Northavon District Council. The group were preceded by the official town-crier from Wotton-Under-Edge who have a colourful touch to the proceedings by appearing in traditional costume and equipped with a large handbell. The other four trees were planted at four separate sites in Frenchay Village. It is hoped that most of these saplings, if not all, will grow and mature to give further pleasure to the generations to come, and remind them of close links forged between Frenchay and Pennsylvania in the 17th Century. It is also a pleasure for us to be reminded in the meantime.

G.T.

**Ham Green Hospital**

*Administration*

Both the Director of Nursing Services (Mr. R. Cooper) and the Administrator (Mr. R. Paynter) have been in their posts for a while now and will be joined on the medical side by Dr. G. R. Burston, newly appointed Chairman of the Ham Green Medical Staff Committee. This completes the management of Unit 2.

*Buildings*

During 1983 we hope to have the new kitchen and dining room completed and this should make available the old dining room for use as a leisure centre and for meetings. This is a much needed facility. At the same time a lot of work has been going on upgrading the boiler house. The prefabricated chimney went up very quickly but we did not expect the old chimney to come down in the same vein, as I understand it is to be taken down brick by brick.

*Education*

(Undergraduate) The presence of Medical Students continues to stimulate us and we continue to have a number of Students from both the United Kingdom and abroad, coming to do their elective with us. Presumably they choose July and August because of the nice weather and lovely surroundings!

*Education*

(Postgraduate) These activities continue to increase in number with a number of symposia being arranged in the forthcoming year and I am always grateful for the interest I get from both General Practitioners and Hospital Colleagues. Part I Membership Course in which many members of the Consultant Staff participate, has been very successful.

*Retirement*

During the coming year we shall be losing at least three of our colleagues, after many years of association with Ham Green. These are Dr. Robert Craig, Consultant Chest Physician, Dr. Francis Page, Consultant Neurologists, and Mr. John Crossley, Consultant Gynaecologist.

At the end of February one of our Nursing Colleagues retired (Mr. Graham Smart) after more than thirty years in the hospital. Originally his time was spent working alongside the Chest Physicians as Charge Nurse of the T. B. Ward. Latterly, he has been Nursing Officer with special responsibility for the Exotic Disease Unit. His wife, was one of our E.C.G. Technicians for nine years.

As correspondent I would appreciate receiving any items of information about Ham Green that may be of interest to the Medchi Journal.

